# Craniocervical Orthosis Produced Using 3D Printing Technology for Anterior Head Tilt Posture and Thoracic Kyphosis: A Custom-Manufactured Device Pilot Application Study

**DOI:** 10.3390/bioengineering13091030

**Published:** 2026-09-04

**Authors:** Buse Akça, Senem Güner, Ahmet Gökhan Acar, Yunis Akkaş

**Affiliations:** 1 Graduate School of Health Sciences Ankara, Ankara University, Ankara 06100, Türkiye; buseakca2000@gmail.com; 2 Department of Orthotics and Prosthetics, Faculty of Health Sciences, Ankara University, Ankara 06100, Türkiye; ahmetgokhanacar@gmail.com (A.G.A.); yakkas@ankara.edu.tr (Y.A.)

**Keywords:** forward head posture, craniocervical angle, thoracic kyphosis, craniocervical orthosis, three-dimensional printing

## Abstract

Forward head posture (FHP) is common in young adults using technological devices and often accompanied by increased thoracic kyphosis. This study reports the design and custom manufacturing of a 3D-printed craniocervical orthosis and a pilot application evaluating its effects on the craniocervical angle (CVA), thoracic kyphosis angle (TKA), and user satisfaction. In this prospective, single-arm, within-subject pilot study, each participant underwent 3D scanning, and a custom orthosis was manufactured from their scan data using a common three-point CAD template via FDM 3D printing. Ten volunteers aged 18–25 years completed the study without a priori sample size calculation. The CVA and TKA were assessed photogrammetrically at baseline and weeks 2, 4, and 6, under orthosis-assisted and non-assisted conditions, with devices iteratively adjusted over six weeks. Satisfaction was evaluated at follow-up completion using the Quebec User Evaluation of Satisfaction with Assistive Technology (QUEST 2.0-TR), and data were analyzed using a linear mixed model. Of the 13 individuals screened, 10 completed the protocol (one discontinued owing to discomfort and two withdrew voluntarily); no serious adverse events occurred. The CVA was significantly higher, and the TKA significantly lower, under the orthosis condition at every time point (all *p* < 0.001), reflecting an acute, device-on effect rather than a demonstrated lasting, unassisted postural correction. Mean device satisfaction was 4.88 ± 0.12/5 and mean service satisfaction was 5.00 ± 0.00/5 among the 10 completers; these scores exclude the participant who discontinued owing to discomfort and two who withdrew for unrelated reasons, which likely inflates the apparent satisfaction level. The custom-manufactured orthosis was feasible to design and fabricate, tolerable after iterative adjustment, and associated with improved craniocervical and thoracic alignment when worn; these findings reflect an acute, device-on effect and do not yet establish lasting postural correction without the orthosis. Given the single-arm, non-blinded pilot design, findings are preliminary and hypothesis-generating, supporting further confirmatory trials.

## 1. Introduction

Correct posture is defined as the musculoskeletal balance that allows the body to function under minimal stress and strain, yet many individuals today fail to maintain ideal postural alignment [1]. With increasing use of technological devices, postural disorders characterized by anterior head positioning have become more common [2]. This condition, forward head posture (FHP) or anterior head tilt posture, is increasingly linked to prolonged smartphone, tablet, and computer use. Anterior head tilt increases cervical vertebral load and, over time, may alter cervical vertebrae, ligaments, tendons, and muscles; head weight (approximately 4.5–5 kg in neutral position) substantially increases spinal load as flexion increases [3].

The craniocervical angle (CVA) is the most widely used parameter for quantifying FHP; a smaller angle indicates greater anterior head displacement, and photograph-based CVA measurement shows high reliability and validity when standardized [4]. FHP-related alignment changes are not limited to the cervical spine and may produce compensatory thoracic changes; the thoracic kyphosis angle (TKA) is therefore frequently assessed alongside CVA [5].

Various approaches manage FHP, including postural education, ergonomic adjustment, deep cervical flexor exercise, and manual therapy [6]. Orthotic interventions—from soft cervical collars to rigid craniocervical orthoses, posterior neck weighting systems, and scapular-retraction trunk orthoses—aim to limit anterior head tilt and increase postural awareness [7]. Craniocervical orthoses have previously produced significant CVA increases even with short-term use [8].

Custom-made, 3D-printed craniocervical orthoses have gained interest for FHP management. Additive manufacturing enables digital scanning and modelling tailored to individual anatomy, allowing more controlled application of corrective forces [9]. Because each device is derived from the wearer’s own scan data, it can achieve a closer, more evenly distributed fit than mass-produced devices, potentially improving comfort and satisfaction [10]. However, studies on the short-term effects of individually manufactured, 3D-printed craniocervical orthoses on FHP and thoracic alignment remain limited.

The novelty of this work is not the use of 3D printing as such, but the way it is applied here: each participant’s orthosis is manufactured individually from their own scan, built on a shared three-point design rather than a single device handed out to everyone, and the fitting process is refined step by step as feedback comes in, with CVA and TKA tracked photogrammetrically throughout. To our knowledge, this combination has not been reported before for FHP-directed craniocervical orthoses. Given the novelty of this three-point-principle design, we adopted a pilot application rather than a definitive trial: each participant received an individually custom-manufactured device built on a common CAD template. Two things were of interest going in. First, whether individualized scanning and manufacturing could actually be carried out reliably, and whether the resulting devices were tolerable once fitted and adjusted. Second, whether the design showed any early signal of an effect on the CVA and TKA, together with how participants rated the device, so that a proper sample size could be worked out for a larger controlled trial afterward.

## 2. Materials and Methods

### 2.1. Study Design

This study was designed and is reported as a prospective, single-arm, within-subject pilot application, in which each participant served as their own comparator across orthosis-assisted and non-assisted conditions. This design was chosen because the orthosis evaluated was a novel, purpose-built device; consistent with recommendations for pilot and feasibility studies, its primary purpose was to establish the feasibility of individualized manufacturing, device tolerability, and a preliminary effect estimate to guide the design and sample size of a future randomized, assessor-blinded controlled trial, rather than to provide a confirmatory efficacy test [11]. Accordingly, no control group, randomization of condition order, or assessor blinding was implemented at this stage.This structure follows from those two aims directly: a single-arm, within-subject design was enough to check feasibility, manufacturability, and tolerability for each custom device, while measuring CVA/TKA repeatedly with and without the orthosis gave us the preliminary numbers needed to plan a properly powered follow-up trial.

The study was approved by the Ankara University Faculty of Medicine Human Research Ethics Committee (Approval No: 2023000769-2 (2023/769); Approval Date: 11 January 2024) and was conducted in accordance with the Declaration of Helsinki. Written informed consent was obtained from all participants. This pilot was not registered in a clinical trial registry, consistent with its exploratory, non-comparative status; findings are intended, in part, to support the registration of a future confirmatory trial.

### 2.2. Participants

This study was conducted at the Ankara University Faculty of Health Sciences, Department of Orthotics and Prosthetics Laboratory, between February 2024 and December 2025. Of 13 participants aged 18–25 years screened, 10 completed the study and were analyzed. Each participant underwent individual 3D scanning for the manufacture of their own custom orthosis (Section 2.4). One participant discontinued, owing to device discomfort, and two withdrew voluntarily before completing follow-up (Table 1); no other adverse events occurred.

### 2.3. CVA and TKA Measurement

CVA, a widely used parameter reflecting craniocervical alignment, was used together with TKA for angular evaluation; both were measured to determine eligibility and repeated at baseline, week 2, week 4, and week 6, under orthosis-assisted and non-assisted conditions. Measurements were obtained with participants standing naturally, in the sagittal plane, from 80 cm. For CVA, the C7 spinous process and tragus were palpated and marked; lateral photographs were taken at shoulder height using the same smartphone camera (Samsung Galaxy A52) for all participants, and the angle between a horizontal line from C7 and the C7–tragus line was measured using the Angulus mobile application by an experienced orthotist. For TKA, the T1, T3, T11, and L1 vertebrae were marked, and the angle was measured similarly. The Angulus application and Kinovea software (used for photogrammetric verification) have separately shown acceptable validity and reliability for angular measurement in other joint/body regions [12,13]; however, a region-specific reliability coefficient for this CVA/TKA protocol was not calculated in the present pilot, a priority for the confirmatory study, particularly because the same assessor applied the orthosis and performed the measurements without blinding. All measurements were performed by the same orthotist using the same device; the order of orthosis/non-orthosis assessment within each visit was not randomized.

### 2.4. Orthosis Design and Custom Manufacturing

**Figure 1:**
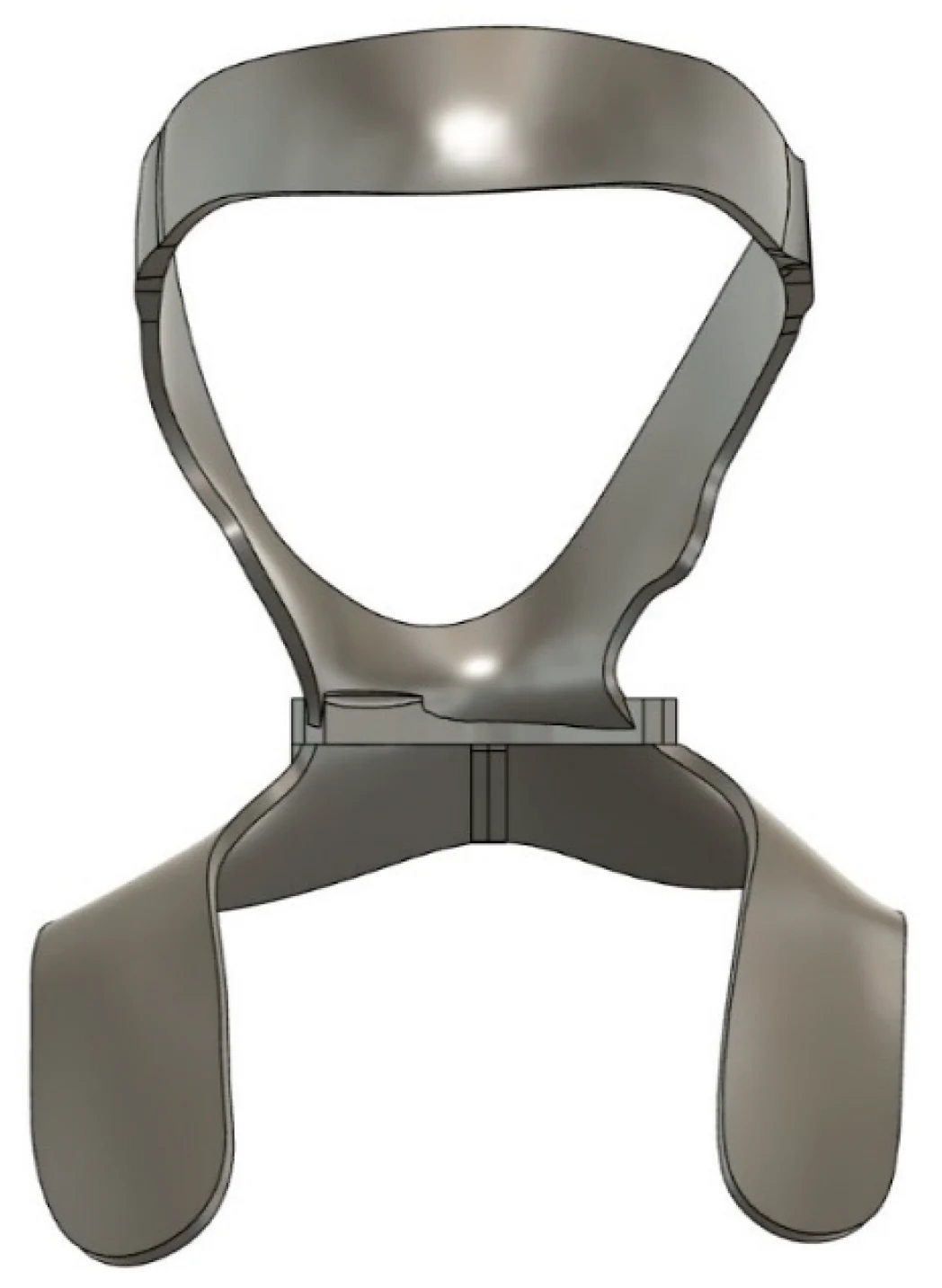
Anterior view of the craniocervical orthosis design (computer-aided design rendering; representative example from the common three-point-principle CAD template used to manufacture each participant’s individually custom-fitted device).

Each participant’s craniocervical orthosis was individually manufactured from that participant’s own 3D scan, obtained with a hand-held tablet scanner while the operator moved around the standing participant at a fixed distance. Scan data were transferred to a computer, where gap-filling, densification, and surface smoothing were performed using Meshmixer and Autodesk Fusion 360. All devices were designed from a common, parametric three-point-principle CAD template—with contact and force application at the frontal region, occiput, and subclavicular/shoulder region—into which each participant’s scan geometry was incorporated before printing. Devices were manufactured on an FDM 3D printer using PLA filament. Thus, while every orthosis shared the same design principle, contact-region layout, and manufacturing protocol, each physical device was custom-fabricated to its wearer rather than being a single, shared device (Figure 1); fit was checked and, where necessary, further individually adjusted (Section 2.6). No mechanical or biocompatibility testing was carried out on the specific filament batch used here. For general orientation, FDM-printed PLA is reported elsewhere in the literature to typically reach tensile strengths around 22–45 MPa and elastic moduli around 2.0–3.2 GPa depending on infill and print settings; these figures are given only as background and are not a characterization of the material actually used in this study.

### 2.5. Orthosis Application

Following postural evaluation, each participant’s custom-manufactured orthosis was fitted, checked, and trialled. Participants were informed about correct donning/doffing, daily use, and recommended wear duration; fitting was demonstrated by positioning the orthosis on the shoulders first, then the head component. Participants were advised to wear the orthosis one hour per day, seated or during an activity, for six weeks total.

### 2.6. Follow-Up and Iterative Fit Adjustment

**Figure 2:**
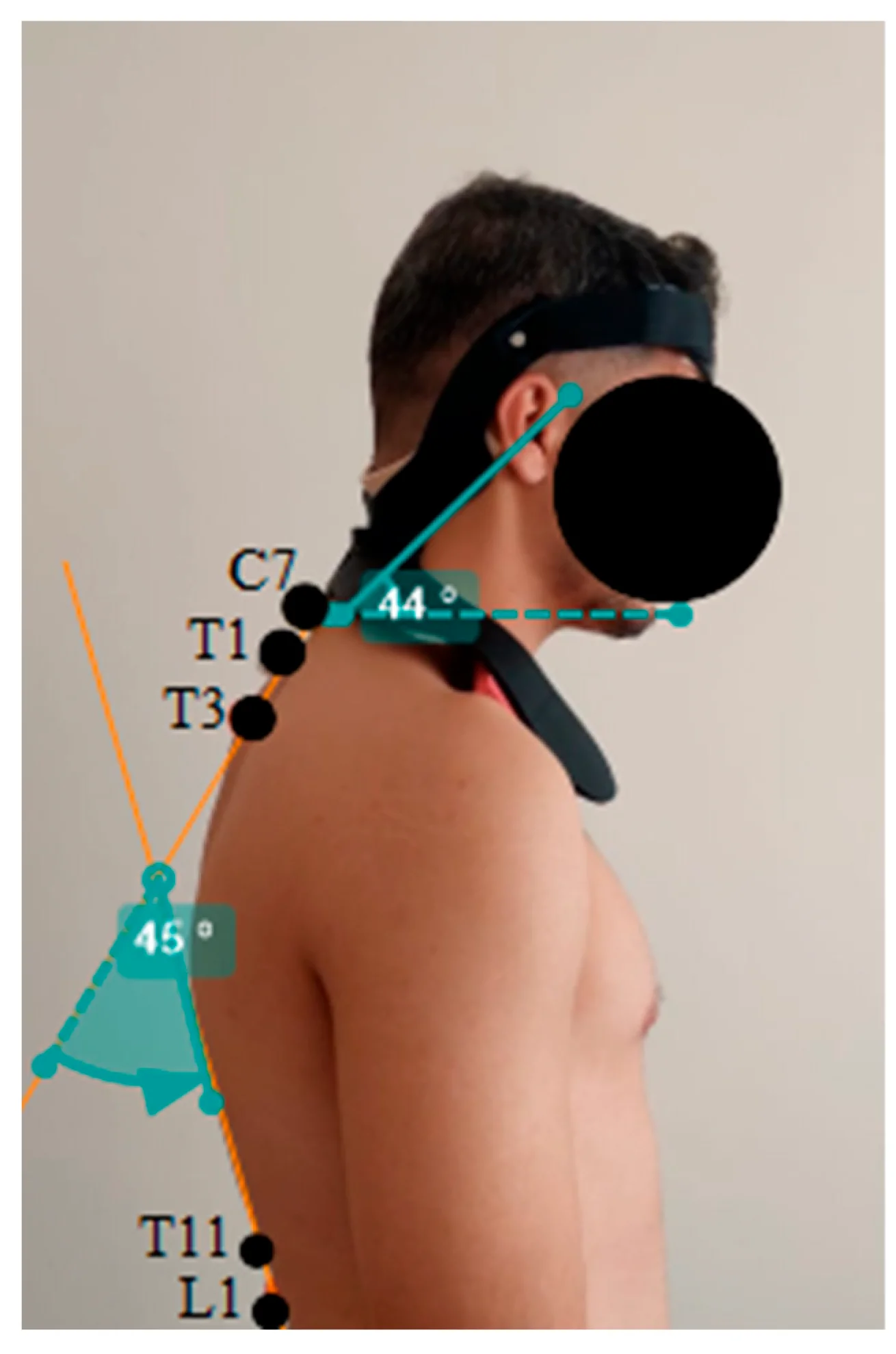
A participant wearing their individually custom-manufactured craniocervical orthosis, with CVA/TKA measurement markers visible.

Participants were followed for six weeks, with control evaluations every two weeks. At each visit, CVA and TKA were measured under both conditions (Figure 2), and structured feedback on discomfort or fit was collected and used to guide the iterative adjustment of each participant’s device. Soft padding was added to the frontal and, where needed, occipital regions in response to reported pressure; the frontal segment was converted to a hook-and-loop closure for a closer fit to each participant’s head circumference. No skin breakdown occurred, and reported pressure and transient erythema resolved with these modifications—a meaningful process outcome of this pilot alongside the clinical data below.

### 2.7. Satisfaction Assessment

To evaluate satisfaction with the custom-manufactured orthosis, the QUEST 2.0-TR was administered at the end of the six-week follow-up. This 5-point Likert-type scale yields mean device (items 1–8) and service (items 9–12) satisfaction scores without a defined cut-off; higher scores indicate greater satisfaction [14,15]. As the questionnaire was administered only at follow-up completion, it was completed only by the 10 participants who reached that time point.

### 2.8. Statistical Analysis

As a pilot application rather than a confirmatory trial, no a priori power calculation was performed; a sample of 10 completers is consistent with pragmatic pilot-study recommendations (approximately 12 participants per arm) intended to generate effect estimates rather than to test a pre-specified hypothesis [11]. Analyses were performed using SPSS (IBM SPSS Statistics, version 27), accounting for the repeated-measures design and small sample size. The Shapiro–Wilk test showed CVA data normally distributed at all time points (*p* > 0.05); week-6 TKA data deviated from normality (*p* < 0.05). A linear mixed model (LMM) was used, with condition (orthosis/no orthosis), time (baseline, week 2, 4, 6), and their interaction as fixed effects and participant as a random effect; reference categories were the orthosis condition and baseline. Significance was set at *p* < 0.05; given the pilot’s exploratory purpose, *p*-values should be interpreted as descriptive of this sample rather than as confirmatory hypothesis tests. Once a paired effect size (Cohen’s dz) can be calculated from individual-level data, it will help set a proper target for the confirmatory trial. In the meantime, and only as a rough planning figure, we take roughly 3–5 degrees of CVA/TKA change as the smallest difference likely to be distinguishable from measurement error with photogrammetric methods; this is a placeholder, not a validated threshold, and should be revisited once an actual MCID is established (Section Limitations). The estimates reported here are meant mainly to feed into that future power calculation.

We did not calculate a between-condition effect size (e.g., Cohen’s d). Because orthosis and non-orthosis measurements came from the same participants, the statistically appropriate effect size is a paired Cohen’s dz, calculated from within-subject differences; only aggregate, condition-level means/SDs were available for this report, not the individual-level differences required. An effect size derived instead from pooled cross-sectional SDs would misrepresent the paired design and could mislead; we therefore omit it and recommend that future analyses of this dataset compute dz directly from individual-level data.

## 3. Results

### 3.1. Participant Flow and Tolerability

Of the 13 individuals screened, 10 (76.9%) completed the six-week protocol and were analyzed (Table 1). No serious adverse events occurred. Transient pressure and mild erythema over the frontal/occipital contact areas, reported by a subset of participants during early follow-up, resolved after the padding and strap modifications described in Section 2.6; no participants discontinued for this reason. One participant discontinued owing to poor device fit, and two withdrew for unrelated reasons. Because QUEST 2.0-TR (Section 3.5) was administered only at study completion, these three participants did not contribute satisfaction data.

### 3.2. Demographic Findings

Demographic and anthropometric characteristics are presented in Table 2. Mean age was 20.4 ± 2.37 years, mean height was 178.6 ± 7.53 cm, mean weight was 74.4 ± 11.35 kg, and mean body mass index (BMI) was 23.20 ± 1.79 kg/m^2^.

### 3.3. CVA Findings

**Figure 3:**
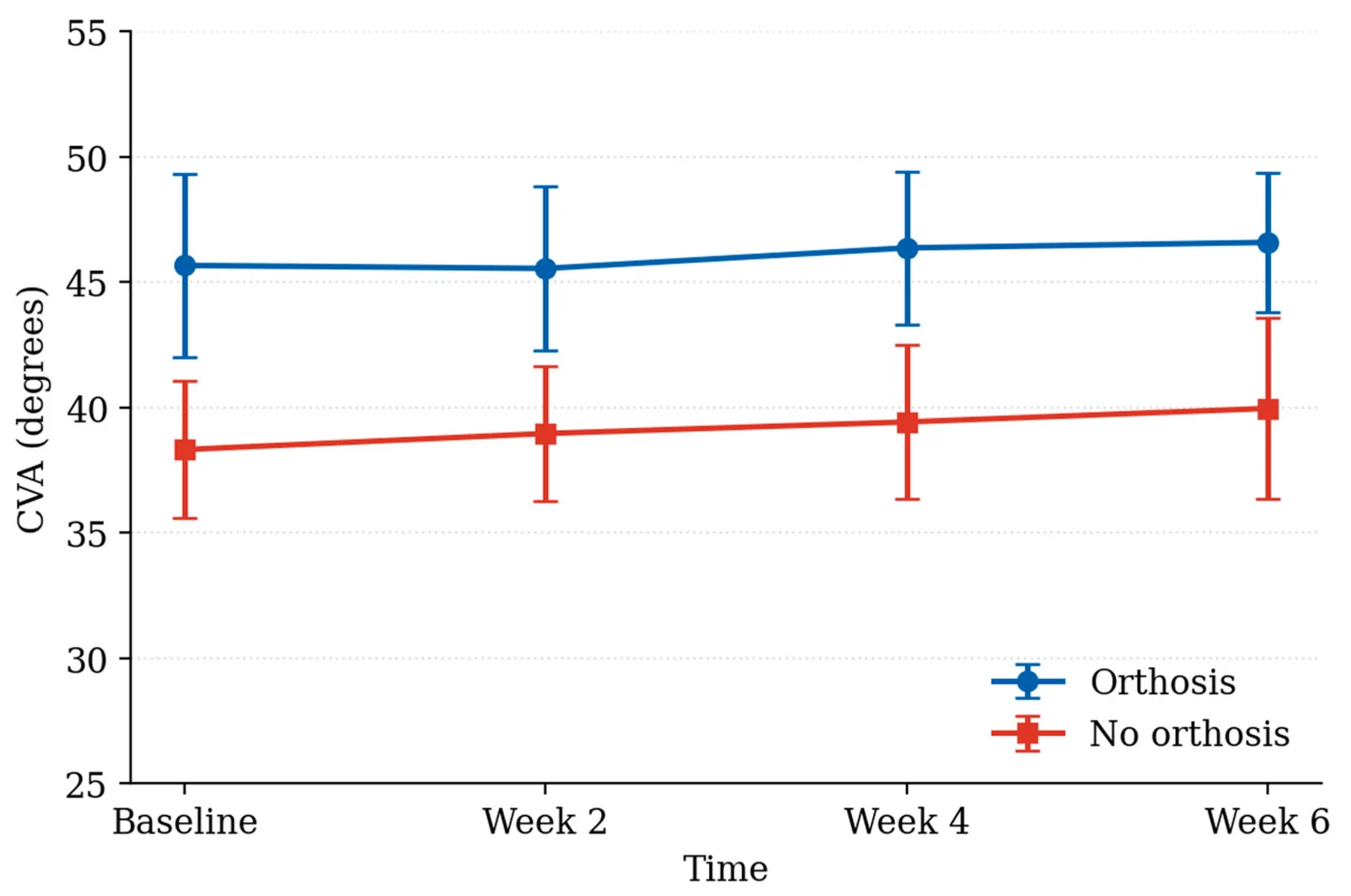
Mean CVA values (±SD) under orthosis and non-orthosis conditions at baseline, week 2, week 4 and week 6.

CVA data followed a normal distribution at all time points (Shapiro–Wilk, *p* > 0.05). CVA values under the orthosis condition were higher those under the non-orthosis condition at every time point, with a slight increasing trend over time under both conditions (Table 3, Figure 3). The LMM analysis (Table 4) indicated that the orthosis condition produced significantly higher CVA values than the non-orthosis condition at every time point (all *p* < 0.001); the within-condition change over time in the non-orthosis group was not significant (*p* > 0.05).

### 3.4. TKA Findings

**Figure 4:**
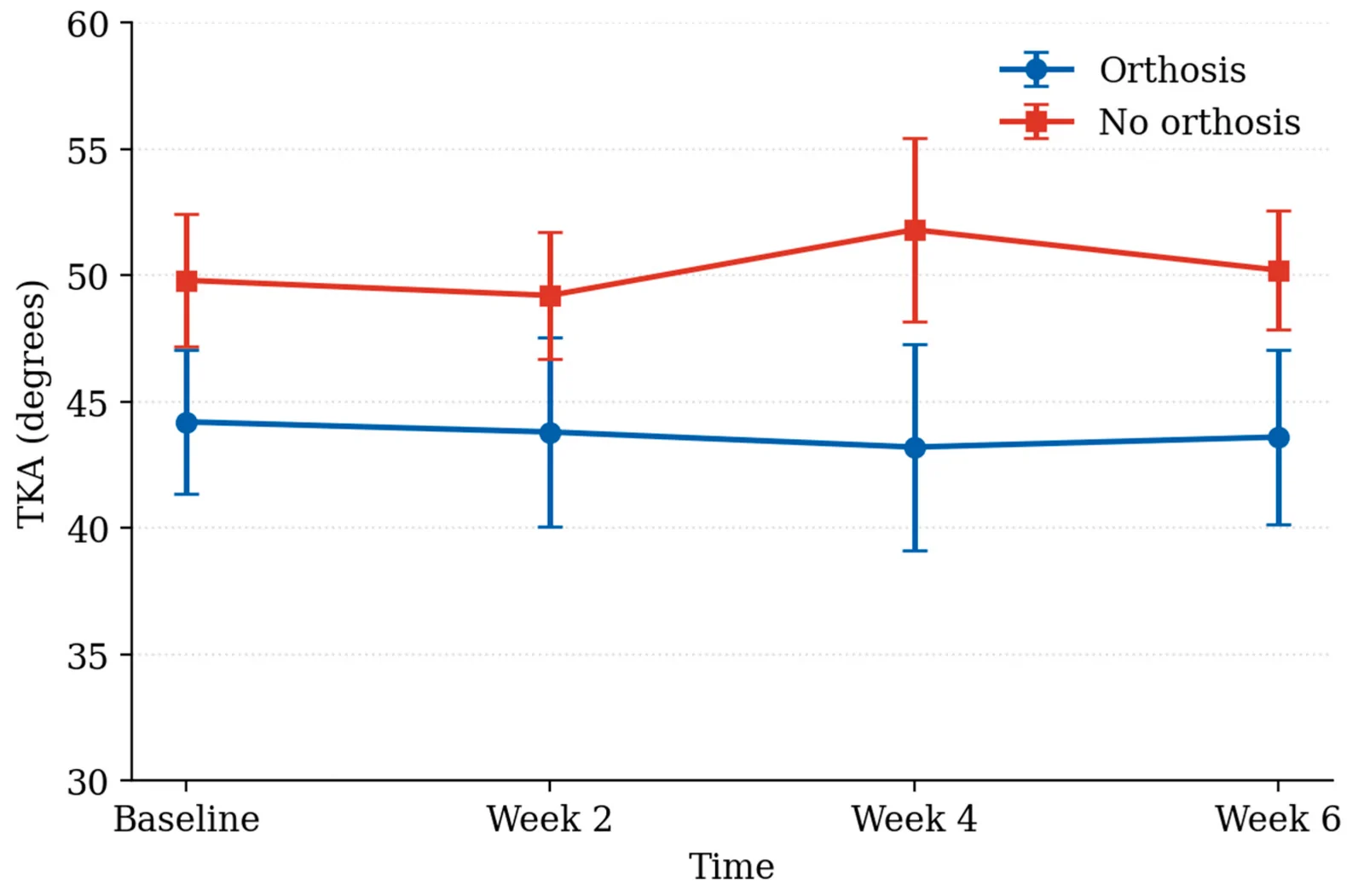
Mean TKA values (±SD) under orthosis and non-orthosis conditions at baseline, week 2, week 4 and week 6.

The LMM analysis indicated that TKA values under the non-orthosis condition were significantly higher than those under the orthosis condition at every time point (all *p* < 0.001; Table 5 and Table 6, Figure 4); the within-condition change over time in the non-orthosis group was not significant (*p* > 0.05).

### 3.5. Satisfaction Questionnaire Findings

Satisfaction scores are presented in Table 7. Mean device satisfaction was 4.88 ± 0.12/5; mean service satisfaction was 5.00 ± 0.00/5, with all participants giving the maximum score. As noted in Section 3.1, these scores reflect only the 10 completers; the participant who discontinued owing to discomfort, and the two who withdrew for other reasons, did not contribute satisfaction data.

## 4. Discussion

This pilot evaluated the feasibility, tolerability, and preliminary effects of an individually custom-manufactured, 3D-printed craniocervical orthosis on the CVA and TKA, together with user satisfaction, in young adults with FHP and increased thoracic kyphosis. As a single-arm pilot without randomization, blinding, or a control group, the findings should be read as preliminary and hypothesis-generating. Within that frame, individualized manufacturing and fitting were feasible, devices were tolerable after iterative refinement, and orthosis use was associated with improved head–neck alignment and a more limited, favourable trend in thoracic kyphosis.

The observed CVA/TKA differences are consistent with the biomechanical principle underlying the three-point design: force balance between the frontal, occipital, and subclavicular regions may oppose anterior head translation, reducing cervical flexion moment and allowing the head’s weight to be carried along a more physiological axis. Three-point craniocervical supports have previously been reported to limit anterior head translation and increase the CVA [16,17,18], consistent with the multi-segmental biomechanics of FHP [2,19]. Because measurements were taken with and without the orthosis in the same session rather than as a true unassisted baseline comparison, the differences reported here should be understood primarily as an acute, device-on effect rather than definitive evidence of lasting change in unassisted posture. The between-condition differences (approximately 6.6–7.3° for CVA, 5.4–8.6° for TKA) appear substantial relative to the standard deviations in Table 3 and Table 5; however, as noted in Section 2.8, we have not converted these into a numerical effect size, because the pooled calculation available from aggregate data risks misrepresenting a repeated-measures comparison. A future paired effect size (Cohen’s dz), calculated from individual-level data, is recommended. A feedback mechanism—continuous head/neck contact increasing postural awareness and discouraging incorrect positioning—has also been proposed [18]; we cannot confirm this from the present data.

The more limited TKA change relative to the CVA is unlikely to reflect thoracic structural rigidity alone: thoracic posture is reported to change more through habitual, long-term adaptation than cervical posture [20], and correcting kyphosis depends on segmental mobility and trunk muscle control, as well as alignment [5]. FHP is thought to arise primarily, with thoracic kyphosis often developing as a secondary, cervicothoracic-junction-mediated adaptation [5,19,21]; correcting cervical alignment alone may therefore have only a limited, indirect thoracic effect [21].

A central contribution of this pilot was demonstrating that individually custom-fabricated devices, built on a common CAD template, could be produced, fitted, and iteratively refined across a cohort: added padding and a hook-and-loop frontal closure, both arising from participant feedback, resolved reported pressure and erythema, illustrating the value of a pilot phase—even with fully custom devices—for refining a design template before a larger trial [10]. The QUEST 2.0-TR results indicate that participants who completed the protocol rated their device and its associated service favourably [14,15]; however, because the questionnaire was administered only at completion, it necessarily excluded the participant who discontinued owing to discomfort—whose assessment would plausibly have been less favourable—and the two participants who withdrew for unrelated reasons. Those three participants amount to nearly a quarter of the 13 originally screened, so what we have is satisfaction data from the people who stuck with the device, not from everyone who tried it. That almost certainly pushes the average up compared with what a full, un-selected cohort would report. The high mean scores should therefore be read as characterizing satisfaction among completers only, not the full range of individuals who might be offered this orthosis.

Measurements were performed under standardized conditions by a single, experienced orthotist, supporting internal consistency; however, because the same individual fitted/adjusted each device and performed all measurements without blinding, and because no formal inter-/intra-rater reliability coefficient was calculated for this specific CVA/TKA protocol, some measurement bias cannot be excluded. Together with the lack of blinding, this is probably the single biggest methodological gap in the present pilot, and closing it—by using an assessor who is not the same person fitting the device, and by conducting formal inter- and intra-rater reliability testing—should be a non-negotiable part of the randomized trial we outline below.

The statistically significant differences reported (all *p* < 0.001) should not be equated with confirmed clinical importance: a validated minimal clinically important difference for the CVA or TKA in orthotic FHP management has not been established. Future confirmatory trials should incorporate patient-reported outcomes (e.g., neck pain/disability indices) alongside angular measurements and paired effect sizes to establish clinical relevance. As noted in Section 2.8, we use roughly 3–5 degrees as a working benchmark for what might be clinically relevant, pending a proper MCID study built around patient-reported outcomes. The differences we observed here—about 6.6–7.3 degrees for the CVA and 5.4–8.6 degrees for the TKA—are above that rough benchmark, which we interpret as encouraging rather than conclusive.

Consistent with reviews emphasizing that single interventions may be insufficient for FHP management [2,7], custom-manufactured craniocervical orthoses such as this one might best be considered part of multicomponent postural rehabilitation rather than a stand-alone treatment. Continuous orthotic support could theoretically reduce muscle activity and promote passive dependency; limiting daily wear to one hour in this protocol reflects an intent to position the device as a temporary, educational tool rather than a permanent aid [22], although this assumption requires explicit future testing.

### Limitations

Several limitations follow from this study’s status as a pilot rather than a confirmatory trial. No a priori power calculation was performed, and the sample of 10 completers cannot exclude false-positive or false-negative findings; the study should not be used alone to establish efficacy. The design lacked a control group, randomized condition order, and assessor blinding, and the same orthotist fitted devices and performed measurements, risking measurement or expectation bias. Orthosis and non-orthosis measurements were obtained in the same session rather than at an independent unassisted follow-up, so differences reflect an acute device-on effect that cannot be fully separated from carry-over or training effects. No region-specific reliability coefficient was calculated for the CVA/TKA protocol. Follow-up was limited to six weeks, precluding conclusions about longer-term effects, adherence, or dependency risk. Although every device was individually custom-manufactured, all were built on a single CAD template and manufacturing protocol, limiting generalizability to alternative designs or materials. A paired effect size (Cohen’s dz) was not calculated because individual-level data were unavailable. QUEST 2.0-TR satisfaction data are subject to attrition/survivorship bias, as they were collected only from the 10 completers, excluding the participant who discontinued owing to discomfort and two who withdrew for unrelated reasons, likely inflating apparent satisfaction. Finally, all participants were young, healthy adults, limiting generalizability to other populations. These limitations directly motivate the design features—randomization, assessor blinding, an independent control condition, a priori power calculation, formal reliability testing, paired effect-size reporting, and non-completer acceptability data—recommended for the confirmatory trial proposed below. A few things were also simply out of scope for this pilot: we did not run mechanical tests on the PLA filament (tensile strength, modulus, hardness, glass transition temperature), did not pursue ISO 10993 [23] biocompatibility testing, and did not build a finite element model of stress at the orthosis or the skin/bone interface [23]. Safety during wear was instead managed in a practical way, by keeping wear time short and checking participants closely every two weeks (Section 2.6). We also did not record EMG or test deep neck flexor strength, so the theoretical worry about muscular dependence discussed above remains untested here. Before the confirmatory trial goes ahead, we intend to bring in an assessor who is blinded to condition and not involved in fitting, randomize the order in which conditions are assessed, add separate visits with a washout period so acute and lasting effects can actually be told apart, run a small reliability sub-study (10–20 healthy volunteers, inter- and intra-rater ICC) before treating CVA/TKA as trial endpoints, follow-up with anyone who drops out for a brief exit interview and shortened satisfaction questionnaire, and get proper mechanical/biocompatibility data on the filament, along with deep neck flexor endurance and EMG measurements.

Balanced against these limitations, methodological strengths include individualized, custom fabrication from each wearer’s own anatomical scan; standardized measurement by a single experienced orthotist; photogrammetric cross-verification with a second software tool; and structured, feedback-driven refinement of the design template during follow-up [16].

In summary, an individually custom-manufactured, 3D-printed craniocervical orthosis is feasible to design and fabricate, can be iteratively refined for tolerability, and is associated with improved head–neck alignment and a favourable thoracic kyphosis trend while worn, with high satisfaction reported by completers. An adequately powered, randomized, assessor-blinded controlled trial, informed by these observations, is recommended before firmer efficacy conclusions can be drawn.

## 5. Conclusions

In this pilot application, a three-dimensionally designed craniocervical orthosis, based on a common three-point-principle CAD template, was individually custom-manufactured for each participant from their own 3D scan, iteratively refined for tolerability, and applied to individuals with anterior head tilt posture and thoracic kyphosis. Preliminary, single-arm findings suggest that orthosis use was associated with improved craniocervical alignment and a favourable thoracic kyphosis angle while worn, and that devices were, after iterative fit adjustment, well tolerated and rated favourably by completers—a finding that should be read alongside the attrition bias discussed in the Limitations section.

Because this was a single-arm, non-randomized, non-blinded pilot without a control group, power calculation, or paired effect-size estimate, these findings are preliminary and hypothesis-generating. They support further investigation of custom-manufactured craniocervical orthoses as a supportive approach in FHP and thoracic kyphosis management—alongside, not instead of, postural education and exercise—and inform the design of a subsequent randomized, controlled, assessor-blinded trial with formal reliability testing, longer follow-up, paired effect sizes, non-completer data, and patient-reported outcomes. Future studies should also compare orthosis designs and manufacturing approaches and examine effects on muscle activity, function, and daily activities.

## Figures and Tables

**Table 1:** Participant flow through the pilot study.

Stage	*n*
Screened for eligibility	13
Discontinued—device discomfort	1
Withdrew voluntarily	2
Completed 6-week protocol and analyzed	10

Inclusion criteria: (1) voluntary participation with signed informed consent; (2) CVA < 43°; (3) TKA > 45°; and (4) age 18–25 years. Exclusion criteria: history of trauma or surgery to the shoulders, head, or cervical/thoracic spine; pain or stiffness affecting the spine, shoulders, or head; neurological disorder; or any other condition affecting the cervical/thoracic region. Age, sex, height, weight, and history of shoulder/head trauma were recorded for all participants.

**Table 2:** Demographic and anthropometric characteristics of the participants (*n* = 10).

Parameter	Mean ± SD
Age (years)	20.4 ± 2.37
Height (cm)	178.6 ± 7.53
Weight (kg)	74.4 ± 11.35
BMI (kg/m^2^)	23.20 ± 1.79

Note. SD: standard deviation; BMI: body mass index.

**Table 3:** Distribution of CVA values under orthosis and non-orthosis conditions at baseline, week 2, week 4 and week 6.

Time	Orthosis (Mean ± SD)	No Orthosis (Mean ± SD)
Baseline	45.66 ± 3.67	38.32 ± 2.74
Week 2	45.54 ± 3.26	38.96 ± 2.68
Week 4	46.36 ± 3.06	39.42 ± 3.08
Week 6	46.58 ± 2.79	39.96 ± 3.62

Note. Values are presented as mean ± standard deviation (SD).

**Table 4:** Linear mixed model (LMM) results for CVA.

Time	Orthosis (Mean)	No Orthosis (Mean)	Difference	*p* (Condition)
Baseline	45.66	38.32	+7.34	<0.001
Week 2	45.54	38.96	+6.58	<0.001
Week 4	46.36	39.42	+6.94	<0.001
Week 6	46.58	39.96	+6.62	<0.001

Note. Values represent estimated means from the linear mixed model. A paired effect size (Cohen’s dz) is not reported; see Section 2.8.

**Table 5:** Distribution of TKA values under orthosis and non-orthosis conditions at baseline, week 2, week 4 and week 6.

Time	Orthosis (Mean ± SD)	No Orthosis (Mean ± SD)
Baseline	44.20 ± 2.86	49.80 ± 2.62
Week 2	43.80 ± 3.74	49.20 ± 2.53
Week 4	43.20 ± 4.08	51.80 ± 3.61
Week 6	43.60 ± 3.44	50.20 ± 2.35

Note. Values are presented as mean ± standard deviation (SD).

**Table 6:** Linear mixed model (LMM) results for TKA.

Time	Orthosis (Mean)	No Orthosis (Mean)	Difference	*p* (Condition)
Baseline	44.20	49.80	−5.60	<0.001
Week 2	43.80	49.20	−5.40	<0.001
Week 4	43.20	51.80	−8.60	<0.001
Week 6	43.60	50.20	−6.60	<0.001

Note. Negative differences indicate a lower thoracic angle under the orthosis condition. A paired effect size (Cohen’s dz) is not reported; see Section 2.8.

**Table 7:** Descriptive statistics for the satisfaction parameters (completers only, *n* = 10).

Parameter	*n*	Mean ± SD
Mean device satisfaction	10	4.88 ± 0.12
Mean service satisfaction	10	5.00 ± 0.00

## Data Availability

The datasets generated and/or analyzed during the current study are available from the corresponding author on reasonable request.
